# Encapsulation of Fatty Acids Using Linear Dextrin from Waxy Potato Starch: Effect of Debranching Time and Degree of Unsaturation

**DOI:** 10.3390/gels11020091

**Published:** 2025-01-24

**Authors:** Huifang Xie, Qingfei Duan, Guohua Hu, Xinyi Dong, Litao Ma, Jun Fu, Yiwen Yang, Huaran Zhang, Jiahui Song, Qunyu Gao, Long Yu

**Affiliations:** 1 Institute of Chemistry, Henan Academy of Sciences, Zhengzhou 450002, China; xiehf0213@hnas.ac.cn (H.X.); duanqf@hnas.ac.cn (Q.D.); malitao@hnas.ac.cn (L.M.); fujun@hnas.ac.cn (J.F.); yangyw@hnas.ac.cn (Y.Y.); zhanghr@hnas.ac.cn (H.Z.); 2 Université de Lorraine, CNRS, LRGP, F-54000 Nancy, France; 3 School of Material Science and Engineering, Zhengzhou University, Zhengzhou 450001, China; 4 College of International Education, Henan Agricultural University, Zhengzhou 450002, China; 19836036918@163.com; 5 School of Food Science and Engineering, South China University of Technology, Guangzhou 510640, China

**Keywords:** liner dextrin, saturation degree, polymerization degree, fatty acids, debranching enzymes, pullulanase, gelatinization

## Abstract

This study investigates the effects of the debranching time of waxy potato starch using pullulanase and recrystallization on particle morphology, debranching degree, and crystal structure. The results demonstrated that after gelatinization and debranching, the surface of the starch crystals became rough and uneven due to hydrolysis, with most particles showing a fragmented surface. The crystalline state was not significantly changed with debranching time. X-ray diffraction analysis revealed no significant differences in the patterns of recrystallized linear dextrin (LD) after various debranching times. Notably, the short-range ordered structure of LD after debranching and recrystallization was more organized than that of the original or gelatinized starch. Additionally, polarized light microscopy showed that the birefringent pattern disappeared as a result of debranching and recrystallization, indicating the breakdown of particle structure, although the overall particle morphology did not change significantly with varying debranching times. Furthermore, linear dextrin derived from starch debranched for 6 h (with pullulanase at 15 μg/g) successfully embedded stearic acid, oleic acid, and linoleic acid, forming a VI-type starch–fatty acid complex.

## 1. Introduction

Starch modifications have long been recognized for their ability to enhance the functional properties of starch-based products, making them more suitable for various industrial applications and offering significant health benefits. The enzymatic debranching and recrystallization of starch can lead to the formation of novel structures that exhibit improved textural properties, increased resistance to retrogradation, and enhanced solubility. These characteristics are especially valuable in the food industry, where they can be used to create low-calorie, high-fiber food products that are also resistant to digestion, contributing to better glycemic control and satiety [1].

While there is growing interest in waxy potato starch and its complexes with fatty acids, a comprehensive understanding of the factors influencing their formation and resulting properties remains limited. This study aims to address this gap by investigating the effects of different debranching times on the properties of waxy potato starch and its subsequent complexation with fatty acids [2]. Through a detailed analysis of the structural and functional changes occurring during this process, this research not only contributes to the scientific understanding of starch modifications but also offers insights into their potential applications in the food industry, with implications for health and nutrition.

Debranched starch, an enzyme-modified starch, forms low-molecular-weight linear short-chain molecules through the selective hydrolysis of a-d-1,6 glycosidic bonds by debranching enzymes like isoamylase or pullulanase [3,4]. The degree of debranching is generally determined by the amount of reducing sugar or the molecular weight distribution of the starch component. Linear dextrin (LD) is usually formed by connecting dozens to hundreds of glucose units through α-(1,4) glycosidic bonds. It differs from amylose in that linear dextrin is a linear polymer in a strict sense. The properties of LD are affected by the preparation process and recrystallization process [5,6,7]. The branched side chains of starch mainly consist of A, B1, B2 and B3 chains, where the A chain DP was <13, the B1 chain DP was in the range of 13–24, the B2 chain DP was in the range of 25–36, and the B3 chain DP was >36 [8,9]. If the hydrolysis time is less than 0.5 h, pullulanase will preferentially hydrolyze the A chain, and since the A chain is located on the outer side of amylopectin, the proportion of the A chain will increase rapidly in a short hydrolysis time [8,9,10]. When the hydrolysis time was extended to 4–12 h, pullulanase began to hydrolyze the long chains, thus increasing the proportion of B chains in the sample [10,11,12]. When the hydrolysis time was further extended to 14 h, the α-1,4 glycosidic bonds in the B chain might also be hydrolyzed by the enzyme, which would further increase the content of the A chain but gradually decrease the content of the B chain [13]. Amylose (extracted from potato) has a high DP value, and is therefore widely used in the investigation of the starch–lipid complex mechanism [14]. Amylose has an advantage over amylopectin in the formation of starch–lipid complexes, mainly because amylopectin contains more side chains and is highly branched, resulting in more steric hindrance, which is not conducive to the formation of complexes with lipids [15,16]. Zhang and Hasjim et al. pretreat starch with different debranching enzymes (pullulanase and isomerase) to reduce that steric hindrance of the branch structure of amylopectin, thereby increasing the complexation degree of the starch and lipid and further reducing the digestibility of starch [17,18,19]. Gelatinized starches are often used in the preparation of V-amylose complexes because they contain sufficient leached amylose for complexation. More studies have shown that the V-amylose complex prepared from gelatinized starch and lipids is anti-digestible. However, there are few studies about the properties of gelatinized debranched–recrystallized waxy potatoes with different degrees of debranching and their complexes with fatty acids.

Waxy potato starch, with its unique properties, such as high amylopectin content and desirable texture, has emerged as a promising candidate for modification studies. Its ability to form complexes with fatty acids, such as stearic acid, oleic acid, and linoleic acid, has been shown to result in the formation of VI-type starch complexes. These complexes are known for their unique crystalline structures and have been linked to various health benefits, including reduced fat absorption and improved cardiovascular health. The formation of these complexes can also lead to the development of novel food ingredients with enhanced functional properties, such as improved texture and stability in processed foods. In this paper, waxy potato starch was used as the raw material, and pullulanase was used to debranch waxy potato starch after gelatinization for different times to obtain LD. The properties of LD were characterized, and the LD was subjected to complexation with stearic acid, oleic acid and linoleic acid, and the crystal form was analyzed, which slowed the digestibility of starch.

## 2. Results and Discussion

### 2.1. SEM Analysis Results

**Figure 1:**
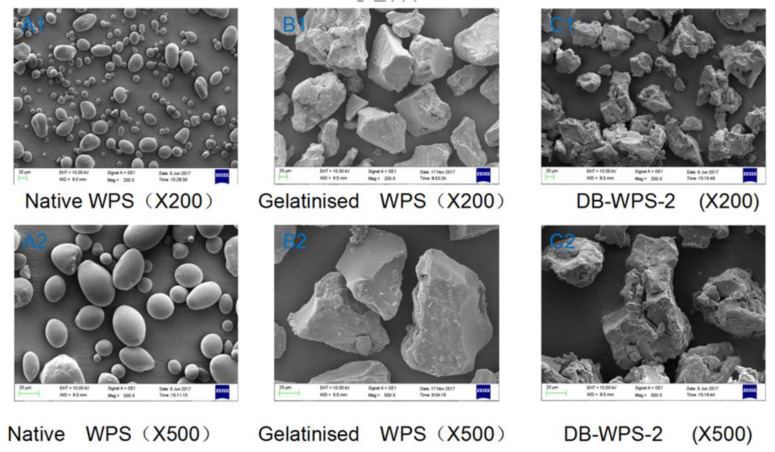

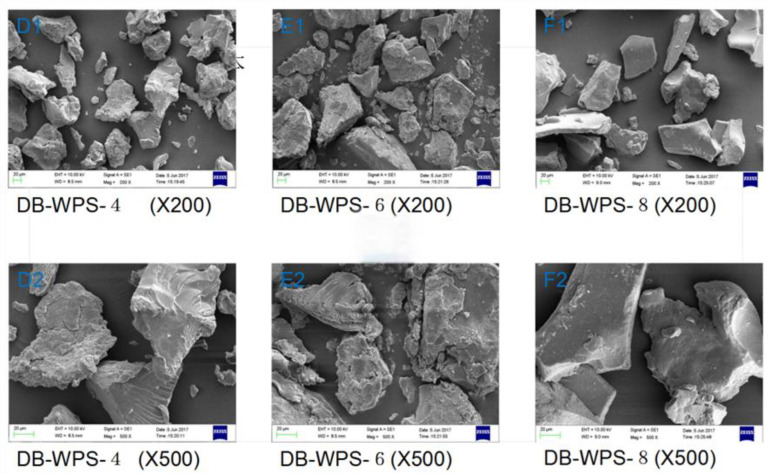
SEM micrographs of native WPS ((**A1**), 200×; (**A2**), 500×), gelatinized WPS ((**B1**), 200×; (**B2**), 500×), DBS-WPS-2 ((**C1**), 200×; (**C2**), 500×), DBS-WPS-4 ((**D1**), 200×; (**D2**), 500×), DBS-WPS-6 ((**E1**), 200×; (**E2**), 500×) and DBS-WPS-8 ((**F1**), 200×; (**F2**), 500×).

SEM was used to observe the micro-morphology and morphology of raw starch, gelatinized starch and debranched starch, as shown in Figure 1. The SEM images showed that debranching was obtained after gelatinization and debranching with pullulans followed by recrystallization. Native waxy potato starch (native WPS), gelatinized waxy potato starch (gelatinized WPS), waxy potato starch debranched for 2 h (DBS-WPS-2), waxy potato starch debranched for 4 h (DBS-WPS-4), waxy potato starch debranched for 6 h (DBS-WPS-6), and waxy potato starch debranched for 8 h (DBS-WPS-8) were used.

It can be found that the grain morphology of debranched starch is significantly different from that of waxy potato starch. α-1,6 glycosidic bonds in starch molecules are hydrolyzed by pullulans, so the branched starch is converted into amylose after shearing and is gradually leached out. The granular structure of starch is destroyed, especially after gelatinization at high temperature. The surface of waxy potato starch granules (Figure 1(A1,A2)) was smooth, and most of them were oval and spherical in shape, with uneven particle size. The particle morphology of the gelatinized waxy potato starch (Figure 1(B1,B2)) is massive and irregular, and the surface of the gelatinized starch particle is relatively smooth. After gelatinization and debranching (Figure 1(B1,B2,C1,C2,D1,D2,E1,E2,F1,F2)), the surface of the recrystallized crystal particle becomes rough and uneven, which indicates that the surface of the particle is hydrolyzed off, and the morphology of the crystal particle after debranching and recrystallization is consistent with the results [20]. For the morphological characteristics of recrystallized debranched starch, the acting force between starch particles is weakened, and the molecules are more dispersed, and the morphology is irregular, which does not change significantly with the change in debranching time. The alpha-1,6 glycosidic bonds in the starch particles can be efficiently and specifically cut off by the starch-debranching enzyme, so that the fragment content of the linear dextrin in the amylolysis product obtained by the amylase is increased. Under specific conditions, free linear dextrin will undergo new changes, such as dissolution, mutual proximity, and molecular chain winding, extension, and even the formation of a double helix, folding and even restructuring to form new crystals.

### 2.2. XRD Analysis Results

**Figure 2:**
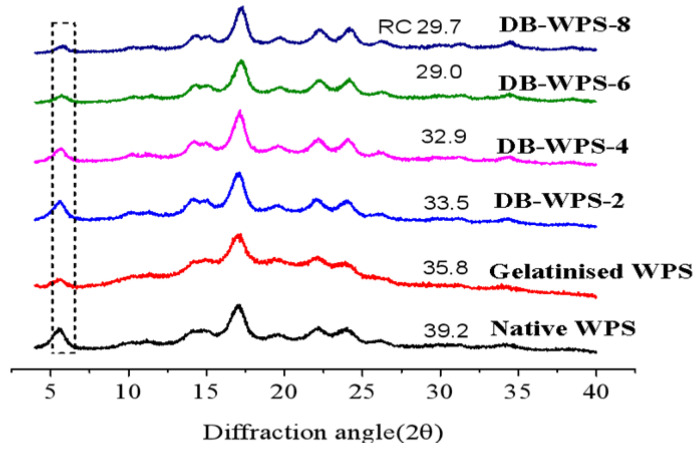
XRD patterns of waxy potato starch debranched by pullulans with different times.

XRD patterns of waxy potato raw starch, gelatinized starch and waxy potato starch with different debranching times are shown in Figure 2. The crystallinity calculated by the X-ray diffraction pattern ranged from 29.7% to 39.2%. It can be seen from the figure that the waxy potato starch has diffraction peaks at 5.6, 17.1, 22.1 and 24.0 (2θ), which are the characteristic diffraction peaks of a typical B-type crystal structure. In addition, the gelatinized starch and the recrystallized starch after debranching for different times also showed B-type crystalline structure, and the typical characteristics of recrystallized or aged starch showed B-type crystalline structure [21]. This is consistent with the results of previous studies [22]. The X-ray diffraction patterns of the recrystallized starches showed slight differences after different debranching times and the crystallinity changed slightly, and the crystallinity was arranged as follows: native WPS (39.2%) > gelatinized WPS (35.8%) > DB-WPS-2 (33.5%) > DB-WPS-4 (32.9%) > DB-WPS-8 (29.7%) > DB-WPS-6 (29.0%).

### 2.3. FT-IR Results

**Figure 3:**
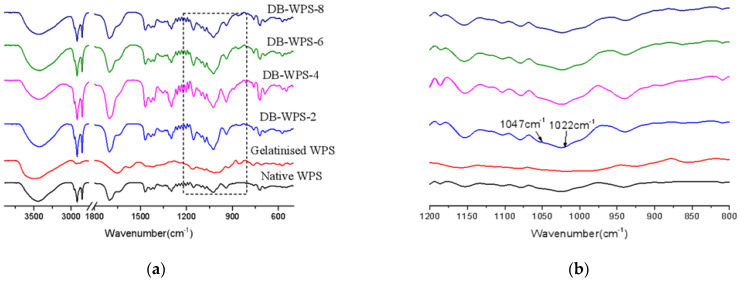
(**a**) FT-IR patterns of waxy potato starch debranched by pullulans with different times, (**b**) enlarged by 1200~800 cm^−1^.

The Fourier-transform infrared spectra of the raw starch, gelatinized starch and waxy potato starch with different treatment times are shown in Figure 3. The FT-IR spectrum is mainly used to measure the short-range ordered structure at the molecular level of starch. The IR spectrum can penetrate into the particle surface at 2 μm below, and further characterize the structure information of the molecular chain structure on the surface of the near-starch particle [23,24]. It can be seen from Figure 3a that the infrared spectra of raw starch and gelatinized starch are different, and the positions of diffraction peaks are basically unchanged from those after debranching recrystallization. It is obvious that the intensity of diffraction peaks of the samples after debranching recrystallization is stronger than that of raw starch. This indicated that the molecular structures of the starch samples were rearranged after the original starch was gelatinized and subjected to debranching recrystallization. The ratio R (1047/1022) of the peak heights occurring at 1047 and 1022 cm^−1^ in Figure 3b is equal to the ratio between the ordered and disordered structures. In combination with Table 1, we can conclude that the deconvolution infrared spectrum of samples with different debranching times changed slightly compared to that of native starch. The ratio of starch samples at 1047 and 1022 cm^−1^ was DB-WPS-4 (1.250) > DB-WPS-6 (1.210) > DB-WPS-2 (1.200) > DB-WPS-8 (1.180) > native starch (1.069) > gelatinized starch (1.039). The results showed that the short-range ordered structure of samples after debranching treatment–recrystallization was more orderly than those of raw starch and gelatinized starch.

### 2.4. ^1^H NMR Spectrum

**Figure 4:**
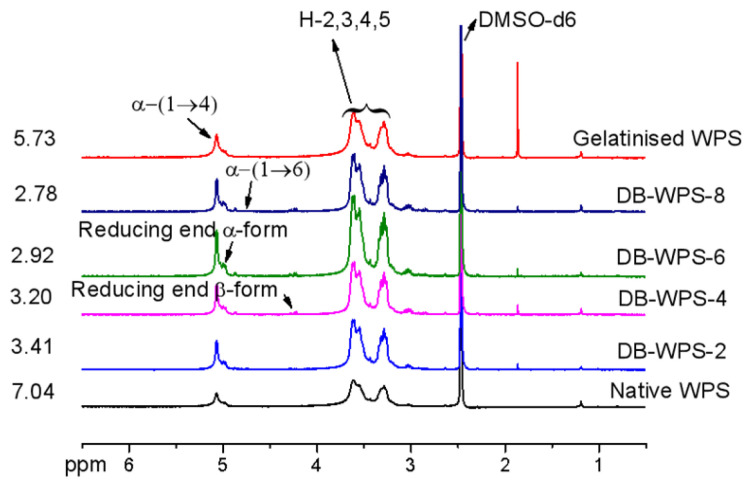
^1^H NMR patterns of waxy potato starch debranched by pullulans with different times.

The ^1^HNMR chromatograms of waxy potato raw starch, gelatinized starch and waxy potato starch with different debranching times are shown in Figure 4. According to Ratnayake et al. [25] and Slveriio et al. [26], the results of the high-performance exchange chromatography determination of the branching degree of amylopectin from different varieties of pea starch were found to be in accordance with the data determined by nuclear magnetic resonance spectroscopy [27]. The result was higher than that of its corresponding normal starch, because the amylose in normal starch mainly contains α-1,4 glycosidic bonds and almost no α-1,6 glycosidic bonds. According to the literature, the chemical offset of α-1,4 glycosidic bonds in the starch molecule was about 5.11, and that of α-1,6 glycosidic bonds was about 4.75. According to NMR software 14.2 integration, the branching degrees of native waxy potato starch, gelatinized starch and debranched starch samples at 2 h, 4 h, 6 h and 8 h were 7.04%, 5.73%, 3.41%, 3.20% and 2.92%, respectively. This indicated that the branching degree of the sample became smaller with the prolongation of the debranching time, reaching a minimum of 2.92% at 8 h. This indicated that with the prolongation of time, more and more α-1,6 glycosidic bonds were cut by pullulanase.

### 2.5. XRD Pattern of Linear Dextrin–Fatty Acid Complex

**Figure 5:**
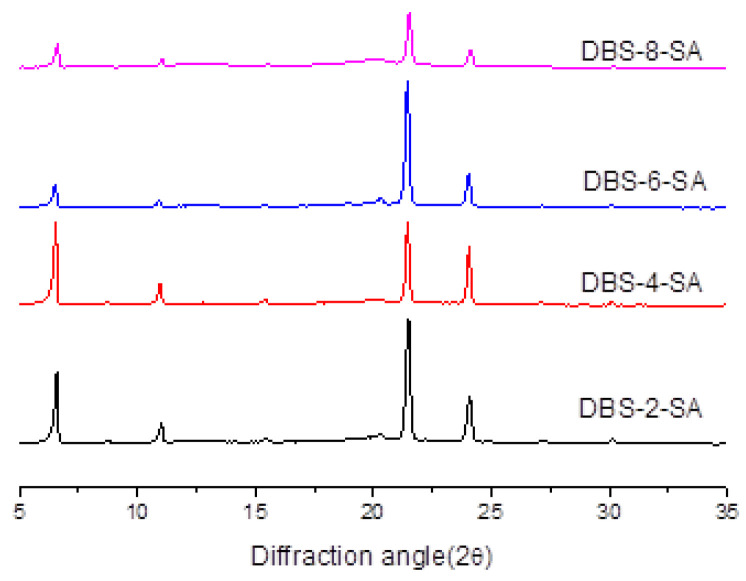
XRD pattern of the complexes of waxy potato starch debranched by 15 μ/g pullulanase for 2 h, 4 h, 6 h and 8 h with stearic acid at a ratio 10:1.

Wide-angle X-ray scattering (WAXS, also known as X-ray diffraction) indicates that the double helix crystallizes into two polymorphs, so-called Form A or Form B. Some plants, such as peas and many other legumes, have granules assigned to a mixed pattern of type C. In the A-type crystal, the double helix is closely packed into a monoclinic cell (dimension A = 20.83 Å, B = 11.45 Å, C = 10.58 Å) containing eight water molecules (IR35M-00023V1, Denver, Germany). In the B-type crystal, the double helix is filled in a hexagonal cell with 36 water molecules (dimension A = B = 18.5 Å, C = 10.4 Å). Complexes of waxy potato starch debranched by 15μ/g pullulanse for 2 h (DBS-2-SA), 4 h (DBS-4-SA), 6 h (DBS-6-SA) and 8 h (DBS-8-SA) with stearic acid at a ratio 10:1 are shown in Figure 5. The relative crystallinity of starch granules varies widely among plant varieties, ranging from 17% to 50%, and waxy starches generally have a higher relative crystallinity than their normal, amylose-containing counterparts. A double helix can be formed between the side chains of amylopectin and distributed in the crystalline area of starch particles. Different arrangements of the double helix structure determine the crystal form of starch. Most of the cereal starch was A-type, the rhizome starch was B-type and the legume starch was C-type [28,29]. Amylose is unstable in aqueous solutions, especially in pure water, and forms a double helix that precipitates easily. These precipitates are crystallites of the polarizing microscope photos [30,31]. Individual amylose molecules also form spirals that easily interact with a series of different compounds, such as iodine, fatty acids or different alcohols, to form complexes. These left-handed helices are more compact than the double helix, and one turn of the helix can contain six to eight glucosyl units, depending on the guest molecule. The individual amylose is spirally crystallized into that so-called V-polymorph [4].

**Figure 6:**
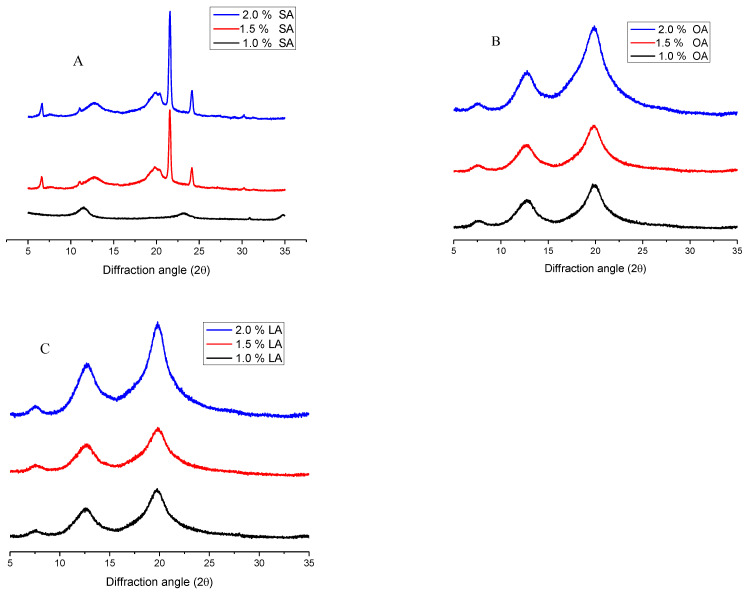
XRD patterns of waxy potato starch debranched for 6 h and (**A**) stearic acid (SA), (**B**) oleic acid (OA) and (**C**) linoleic acid (LA) complexes.

The X-ray diffraction pattern of the waxy potato debranched by pullulans with the 15 μ/g amount for 2 h, 4 h, 6 h and 8 h of the debranched starch/stearic acid ratio 10:1 complex is shown in Figure 6. From Figure 6, the dispersion diffraction peak of DBS-6-SA was the strongest, and the distribution of the X-ray diffraction peak was the closest to the peak type of the V-shaped crystal. Therefore, the debranched potato starch with the 6 h debranching time was selected to react with stearic acid, oleic acid and linoleic acid and to prepare the complex [32].

The X-ray diffraction peak distribution and relative crystallinity of the starch–lipid complex sample were related to the type of fatty acid. The X-ray diffraction diagram of the complex of stearic acid, oleic acid, linoleic acid and debranched starch is shown in Figure 6. As shown in Figure 6, as for stearic acid, the X-ray diffraction diagram of debranched–recrystallized starch shows a B-type crystal structure. When 1% stearic acid is added, the complex has relatively strong diffraction peak intensities at 13.1 and 23.0 (2θ). When 1.5% and 2.0% stearic acid are added, LD and stearic acid form a typical B+V-type crystal structure complex (the diffraction peak intensities are relatively strong at 5.7°, 7.3°, 13.1°, 17.2° and 20.0° at 2θ). The starch changes from a B-type crystalline structure to a V-type crystalline structure. At the same time, when 1%, 1.5% and 2.0% linoleic acid were added, the linear dextrin and linoleic acid formed a V-type crystal structure (with strong diffraction peak intensity (7.3°, 12.9° and 20.0° at 2θ) [33]. The position of the crystallization peak did not change, but the peak intensity increased with the increase in the amount of linoleic acid added. When oleic acid was added, similar results were observed with the addition of linoleic acid. When amylose is in excess of solvent, it is either in a dynamically changing state of random crimp or combined with another amylose to form a double helix [34]. When the appropriate guest was present in the system, the helix structure of amylose and the guest were compounded through hydrophobic interaction [35], maintaining a relatively stable state [36]. Many ligands may be associated with amylose forming a complex. Their existence will cause conformational changes, including the conversion of an amylose double helix to a single helix. The so-called V-amylose thus produced is compact and has a central hydrophobic cavity in which the hydrocarbon chains of the ligands can reside [37,38], and each helix is composed of 6~8 glucose units.

Amylose complexes can be divided into two separable states: the less-ordered form VI-type and the semi-crystalline VII-type amylose complex. The form VI complex is formed at or below 60 °C and results in the formation of randomly oriented individual helical segments. The low temperature results in a high nucleation rate, causing the spirals to freeze rapidly to their positions, resulting in low crystallinity, if any. These complexes dissociate between 95 °C and 105 °C. In another aspect, the form VII complex is obtained by heating a mixture of amylose and a ligand at a relatively high temperature (at least 90 °C) [39]. Under these conditions, the nucleation rate is low, allowing adequate propagation. This results in the structure having distinct crystalline regions [35]. Judging from this, the starch–lipid complex was a form VI complex based on the preparation process (prepared at 60 °C) and the X-ray diffraction pattern.

## 3. Conclusions

In this study, the effects of pullulanase-catalyzed debranching and recrystallization of waxy potato starch were investigated. The results demonstrated that after debranching, the starch particles’ surface became rough and uneven due to hydrolysis, although the overall particle morphology did not undergo significant changes with varying debranching times. X-ray diffraction (XRD) analysis revealed that the recrystallized starch maintained a B-type crystalline structure, with slight variations in crystallinity, and the short-range ordered structure of the debranched starch was more organized than that of native or gelatinized starch. Polarized light microscopy further confirmed that the birefringent pattern disappeared upon gelatinization and debranching, with the starch particles becoming fragmented. Furthermore, the linear dextrin derived from starch with a 6 h debranching time successfully formed a V-type starch–fatty acid complex with stearic acid, oleic acid and linoleic acid. These findings suggest that pullulanase-mediated debranching and recrystallization significantly altered the structural properties of waxy potato starch, enhancing its potential for forming stable starch–fatty acid complexes. This study provides valuable insights into the structural modifications of starch and its interaction with lipids, which could lead to the development of novel starch-based materials with applications in food and other industries.

To scale up the findings, future work could focus on optimizing the debranching and recrystallization process for industrial applications, especially in functional food and material design. Further investigation into complementary fatty acids or alternative starch sources could help refine the starch–lipid complexation process, broadening its application potential.

## 4. Materials and Methods

### 4.1. Materials and Methods

Waxy potato starch of food grade was purchased from AVEBE, Veendam, the Netherlands. Pullulanase, also of food grade, was obtained from Guangzhou Yulibao Biotechnology Co., Ltd., Guangzhou, China. Anhydrous sodium acetate, classified as analytically pure, was provided by Tianjin Damao Chemical Reagent Factory, Tianjin, China. Glacial acetic acid, suitable for analytical purposes, was supplied by Shanghai Runjie Biochemical Reagent Co., Ltd., Shanghai, China. Absolute ethyl alcohol with a purity of ≥99.7% was acquired from Tianjin Fuyu Fine Chemical Co., Ltd., Tianjin, China.

### 4.2. Experimental Methods

#### 4.2.1. Preparation of LD

Waxy potato starch (15 g, db) was dispersed in 0.2 M acetic acid–sodium acetate buffer solution adjusted to pH 5.2. The mixture was stirred continuously to prepare a 10% (*w*/*v*) starch suspension. This suspension was then subjected to continuous stirring using a magnetic stirrer (model IR35M-00023V1, Denver, Passau, Germany) in a boiling water bath for 30 min to achieve complete gelatinization. Subsequently, the temperature of the starch suspension was reduced to 58 °C, corresponding to the best performance for the enzyme used, and this temperature was maintained throughout the subsequent steps. Pullulans at a dosage of 15 U/g, based on the dry starch basis, were introduced to the gelatinized starch suspension to debranch the starch. The enzymatic treatment was carried out for 2, 4, 6 and 8 h, respectively, in a constant-temperature water bath (model HH-2, Jin Tan Fu Hua, Jintan, China). Upon completion of the enzymatic reaction, the suspension was heated to 100 °C for 30 min to inactivate the enzyme. The treated suspension was then cooled to room temperature in an ice-water bath, transferred to a refrigerator, and stored at −20 °C for 48 h. Afterwards, the sample was tempered in an ice-water mixture to room temperature to facilitate the subsequent filtration process, and cold water was added until the ice had completely melted. The sample was then subjected to suction filtration, and the filtrate was lyophilized using a freeze-dryer (model Scientz-18N, Xinzhi Biological, Ningbo, China) to yield linear dextrin [40].

#### 4.2.2. Microscopic Morphology Observation of LD

A few of the starch samples, debranched for different lengths of time, were placed on a metal sample table, and the non-adsorbed floating particles were blown away with a rubber pipette bulb. The samples were gold-sprayed under vacuum for 80 s, and then observed under a scanning electron microscope (EVO18, Zeiss, Oberkochen, Germany), and scanning electron microscope images were obtained.

#### 4.2.3. X-Ray Diffraction Analysis

The X-ray diffraction pattern of the samples was determined by an X-ray diffractometer (D8 ADVANCE, Bruker, Karlsruhe, Germany) [41]. The specific detection conditions were as follows: the scanning step size was 0.05°; the scanning range was 2θ = 4–40°; integration time was 2 s; and scanning speed was 5°/min. Target type: Cu; pipe flow: 26 mA; pipe pressure: 44 KV.

The relative crystallinity of starch granules can be calculated according to the methods of Nara and Komiya [42]. The crystallization area and the total area interval were integrated using Jade 6.0 software, respectively. The obtained ratio was relative crystallinity (RC): the relative crystallinity of starch was calculated by the ratio of the crystallization area to the total diffraction area.

(1) $$\mathrm{RC}\;(\%)=\times 100\%\;\frac{A_{c}}{A_{c}+A_{\alpha }}$$

In Formula (1), RC represents the relative crystallinity; $A_{c}$ represent that area of the crystalline region; and $A_{\alpha }$ represents the amorphous region area.

#### 4.2.4. ATR-FTIR Test

The short-range structure of the sample can be analyzed by attenuated total reflection (ATR) mode in the Fourier-transform infrared (FT-IR) spectrum (Bruker Tensor-27, Germany). The specific conditions for detection were as follows: the resolution was 4 cm^−1^, and the wavelength was scanned in the range of 4000–400 cm^−1^ 16 times [43]. Then, the spectrum was subjected to correction analysis by baseline and deconvolution in the range of 1200–800 cm^−1^. The processing software used in the convolution diagram was OMNIC 6.2, and the corresponding half-peak width was 22 cm^−1^, with a resolution enhancement factor of 2.2. The absorbance amplitudes of the samples at 1022 and 1047 cm^−1^ were recorded, and the ratio R (1047/1022) between 1047 cm^−1^ and 1022 cm^−1^ was calculated based on the absorbance amplitudes to obtain the short-range ordered structure of the starch sample [44].

#### 4.2.5. ^1^H NMR Test

Each 5 mg sample of native starch, gelatinized starch, and debranched starch was accurately weighed and dissolved in DMSO-d6. And approximately 2 mg of deuterated trifluoroacetic acid was added prior to testing. The detection conditions of ^1^H NMR [45] spectroscopy (DMX 400, Bruker, Germany) were as follows: pulse angle of 30°, relaxation time of 10 s, detection time of 2 s and detection temperature of 80 °C. According to the existing literature, and the positions of the characteristic absorption peaks in the NMR spectra of raw starch and debranched starch, in general, the proton characteristic peak connected with α-1,6 glycosidic bond appeared at about 4.75 ppm, and correspondingly, the chemical shift of the proton characteristic peak connected with α-1,4 glycosidic bonds appeared at about 5.11 ppm. The branch degree (DB) of starch can be calculated based on the peak position and peak area information in the NMR spectrum of starch. The formula used is as follows:

(2) $$\mathrm{DB}=\frac{I_{\alpha -1,6}}{I_{\alpha -1,4}+I_{\alpha -1,6}}\times 100\%,$$

In Equation (2), DB refers to the branching degree of starch, $I_{\alpha -1,4}$ is an integral signal of a proton characteristic peak connected by alpha-1,4 glycosidic bonds; and $I_{\alpha -1,6}$ is the integral signal of the proton characteristic peak connected by α-1,6 glycosidic bonds.

#### 4.2.6. Optical Microscope Observation

First, samples of around 50 mg were prepared in suspension with deionized water for 5 min to create a state of uniform mixing. During the test, a small amount of suspension was sucked up by a pipette and placed on the slide, and the cover glass was covered. According to the requirements, the morphology of the samples can be observed and photographed under fields of 200-times and 500-times magnification under ordinary light (EVO18, Zeiss, Germany) and polarized light (Model BHS-2, Olympus, Tokyo, Japan), respectively.

#### 4.2.7. Preparation Method of Linear Dextrin-Lipid Complex

Linear dextrin (3 g) with 40 mL (75 mg/mL) of KOH solution was mixed, stirring at high speed (IR35M-00023V1, Denver, Germany) at 90 °C, and 60 mL (5 mg/mL) of the preheated 90 °C KOH solution with stearic acid (1%, 1.5%, 2%), oleic acid (1%, 1.5%, 2%) and linoleic acid (1%, 1.5%, 2%) was added and stirred for 30 min, respectively. The system was then cooled to 60 °C and 12 mL of 0.1 mol/L HCL solution was added, and the mixture was stirred at 60 °C for 30 min, allowed to stand at 60 °C for 30 min and allowed to cool to room temperature. The system was centrifuged (3-30KS, Sigma, Osterode, Germany) to room temperature, and freeze-dried (Scientz-18N, Xinzhi, Ningbo, China) to obtain the compound [46].

#### 4.2.8. Statistical Analysis of Data

The experimental data were repeated three times, and the standard deviation of the average value was displayed. *p* < 0.05 indicated significant difference.

## Figures and Tables

**Table 1:** The ratio R (1047/1022) of the peak heights occurring at 1047 and 1022 cm^−1^.

Starch Sample	1047/1022
Native WPS	1.069
Gelatinized	1.039
DB-WPS-2	1.200
DB-WPS-4	1.250
DB-WPS-6	1.210
DB-WPS-8	1.180

## Data Availability

The original contributions presented in this study are included in the article. Further inquiries can be directed to the corresponding authors.
